# Fractographic study of the behavior of different ceramic veneers on full coverage crowns in relation to supporting core materials

**DOI:** 10.4317/jced.51293

**Published:** 2013-12-01

**Authors:** Ignacio Farga-Niñoles, Rubén Agustín-Panadero, Juan L. Román-Rodriguez, María F. Solá-Ruíz, María Granell-Ruíz, Antonio Fons-Font

**Affiliations:** 1 DDS, Occlusion and Prosthodontics, Department of Stomatology, University of Valencia, Valencia, Spain; 2 DDS, PhD, Associate professor, Occlusion and Prosthodontics, Department of Stomatology, University of Valencia, Valencia, Spain; 3 MD, DDS, PhD, Adjunct Lecturer, Department of Stomatology, University of Valencia, Valencia, Spain; 4 MD, DDS, PhD, Professor of Occlusion and Prosthodontics, Department of Stomatology, University of Valencia, Valencia, Spain

## Abstract

Objectives: To observe porcelain veneer behavior of zirconia and metal-ceramic full coverage crowns when subjected to compression testing, comparing zirconia cores to metal cores. Study Design: The porcelain fracture surfaces of 120 full coverage crowns (60 with a metal core and 60 with a zirconia core) subjected to static load (compression) testing were analyzed. Image analysis was performed using macroscopic processing with 8x and 12x enlargement. Five samples from each group were prepared and underwent scanning electron microscope (SEM) analysis in order to make a fractographic study of fracture propagation in the contact area and composition analysis in the most significant areas of the specimen. Results: Statistically significant differences in fracture type (cohesive or adhesive) were found between the metal-ceramic and zirconia groups: the incidence of adhesive fracture was seen to be greater in metal-ceramic groups (92%) and cohesive fracture was more frequent in zirconium oxide groups (72%). The fracture propagation pattern was on the periphery of the contact area in the full coverage crown restorations selected for fractographic study. Conclusions: The greater frequency of cohesive fracture in restorations with zirconia cores indicates that their behavior is inadequate compared to metal-ceramic restorations and that further research is needed to improve their clinical performance.

** Key words:**Zirconia, zirconium oxide, fractography, composition, porcelain veneers, fracture, cohesive, adhesive.

## Introduction

The arrival of new dental materials and treatment possibilities generates high esthetic expectations among patients. While restorative dentistry should supply satisfactory esthetics, it must first meet functional needs. Recent research (1,2) and the development of new techniques have lead to the introduction of new ceramic materials for use in prosthodontics and these include zirconia, a material which aims to combine strength and esthetics in restoration techniques.

Numerous clinical studies show that the fracture rate of porcelain veneers varies between 6% and 15% over 3-5 years in restorations with zirconia cores (3-5), while this is only 4% or more over ten years for conventional metal-ceramic restorations (6-8).

The aim of the present study was to analyze the fractures produced in ceramic veneers by static load (compression) testing of full coverage crown restorations, whereby the study variable was the supporting core material, comparing metallic and zirconia cores. The study set out, firstly, to classify the type of fracture produced (adhesive or cohesive) by means of stereomicroscope observation (9,10) and secondly, the form of fracture propagation in the contact area was observed using scanning electron microscope (SEM) fractography (11). Lastly, composition analysis was carried out in order to obtain more information about the processes involved in porcelain veneer fracture.

## Material and Methods

The restorations used in this study were fabricated on the basis of a master cast, which took the form of a maxillary first molar of conventional shape, to obtain a full-coverage fixed crown. One hundred and twenty impressions were taken from the master cast using addition silicone (polyvinyl siloxane) of heavy consistency and silicone fluid (Putty and Light Elite HD®, Zhermack, Italy) using the double-mix technique. Each impression was then cast in epoxy resin (Exakto-Form®, Bredent, Germany). After a 45-minute polymerization, each epoxy resin specimen was removed from the mold and mounted in a 22-mm-diameter copper cylinder, setting the specimen in type IV dental plaster (Pastel Rock Die Stone®, Kerr, Italy).

One hundred and twenty crowns were fabricated and divided into six groups: Group 1 = 20 IPS e.max ZirCAD crowns (core: IPS e.max ZirCAD®, Ivoclar Vivadent, Schanne, Liechtenstein with porcelain veneer: IPS e.max Ceram®, Ivoclar Vivadent); Group 2 = 20 IPS e.max ZirPress crowns (core: IPS e.max ZirCAD® with porcelain veneer: IPS e.max ZirPress®, Ivoclar Vivadent); Group 3 = 20 Lava crowns (core: Lava Frame Zirconia®, 3M ESPE, USA; porcelain veneer: Lava Ceram®, 3M ESPE); Group 4 = 20 metal-ceramic crowns with porcelain stratification layering (core: Rexillium V® nickel-chromium alloy, Pentron Laboratory Technologies, CA, USA, with porcelain veneer: IPS d.SIGN® ceramic, Ivoclar Vivadent); Group 5 = 20 metal-ceramic crowns with porcelain stratification layering (core: Rexillium V® nickel-chromium alloy, Pentron Laboratory Technologies; porcelain veneer: IPS InLine® ceramic, Ivoclar Vivadent); Group 6 = 20 metal-ceramic crowns with heat press ceramic (core: Rexillium V® nickel-chromium alloy, Pentron Laboratory Technologies, with porcelain veneer: IPS InLine PoM® ceramic, Ivoclar Vivadent).

- Morphology/Design Characteristics of the Internal Cap 

The design morphology of each crown, whether with metallic or zirconia core, followed the anatomical design referred to earlier.

The internal crown cap was characterized by two inclined cuspal planes which allowed the porcelain veneer equal thickness over the entire crown surfaces. In the cervical area, the coping was precisely adjusted to the edge of the restoration piece.

- Characteristics of Ceramic Veneer Morphology Design

The occlusal anatomy of each crown was designed using the wax-up technique, so that the load applicator of the Instron machine used for the compression tests (a 4-mm aluminum ball) made contact in the fossa of the restoration with three-point contact on the internal slopes of the vestibular cusps and palatine cusp. To do this, a reproduction of the active part (antagonist) of the load applicator was fabricated by taking an impression using addition silicone (Elite HD®, Zhermack, Italy), which was then cast in acrylic resin (Trim II®, Bosworth).

- Bonding Crowns to the Casts

Once fabricated, the crowns were bonded using a dual-polymerization composite resin cement. A 1-kg force was applied for 5 minutes to ensure correct distribution of the bond material and to seat the crowns properly.

- Compression Testing

The compression test was carried out using a mechanical testing machine (Instron® model 4202, MA, US). The load applicator descended onto the sample exercising continuous vertical force with a crosshead speed of 0.5 mm/s, moving vertically downward perpendicular to the occlusal plane. The load force applicator’s aluminum ball established three-point contact with the internal slopes of the crown’s vestibular cusps and palatine cusp. The machine was stopped once the veneering ceramic had fractured, and the force that had provoked the fracture was measured in Newtons.

- Image Processing of Test Samples

Firstly, all specimens were observed under an optical stereo microscope (Leica APO MZ®, Leica Microsystems, IL, USA) with 8x and 12x enlargement, identifying the type of fracture produced in each sample, classifying these as either cohesive (fracture situated within the internal structure of the porcelain veneering (chipping)), adhesive (at the porcelain veneer-zirconia interface), or complete (complete fracture of the crown). Complete fractures were excluded from the study. Samples were examined on the external surface as well as within the fracture to examine its internal structure.

Sample dimensions were conditioned to analyze their external surfaces. The copper cylinder that held the stump of each sample was sectioned with a 50A15 Struers diamond cutting disk to leave a ring with a depth of 4 mm, using a Secotom-15® machine (Struers, Willich, Germany) at 3,000 rpm and progression of 0.25 mm/min with refrigeration by low-velocity water to avoid damaging the samples.

When the first observation phase was complete, 30 specimens were selected randomly, five from each group, to perform fractography analysis and then composition analysis using SEM. To do this, the surface of each sample was cleaned, eliminating any microparticles, first manually and then using an ultrasonic cleaner (Ultrasonic Cleaner TSD-J 0,7L®, PCE Instruments, Albacete, Spain). Then the sample was placed in a JP Selecta oven model 210 (Abrera, Spain) for 30 minutes at 65º C in order to eliminate surface dampness before metalizing the samples with gold for SEM observation (JEOL JSM 6300 with crystal microanalysis Oxford Instruments Ltd, Tokyo, Japan).

Lastly, the data obtained were submitted to statistical analysis (Bonferroni bivariate analysis). The significance level established for bivariate analysis was 5% (P < 0.05).

## Results

**Figure 1:**
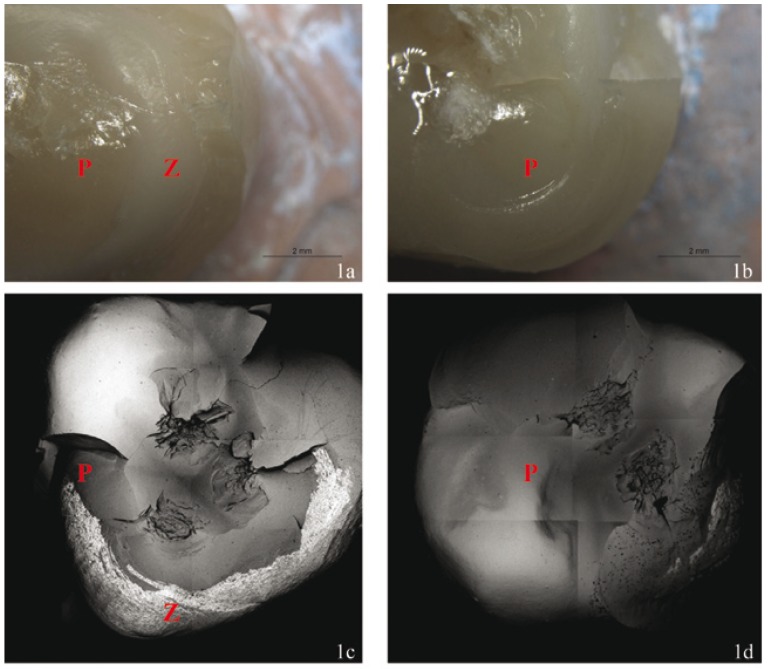
A.-Adhesive fracture of Specimen 8, Group 1 observed under the Stereo Microscope 8x. B.-Cohesive fracture of Specimen 18, Group 2 observed under the Stereo Microscope 8x. C.-Adhesive fracture of Specimen 8, Group 1 observed using SEM BSE x25. D.- Cohesive fracture of Specimen 18, Group 2 observed using SEM BSE x25. P = Porcelain Veneer. Z = Zirconia.

Of the 120 crowns studied, 58.3% suffered adhesive fracture, 40% cohesive fracture, and 1.7% complete crown fracture. In Group 1, 60% of crowns underwent cohesive fracture, 30% adhesive fracture, and 10% complete crown fracture (Fig. 1). In Group 2, 85% of crowns suffered cohesive fracture while 15% suffered adhesive fracture (Fig. 1). In Group 3, 70% of crowns underwent cohesive fracture, while 30% suffered adhesive fracture. In Group 4, all fractures were adhesive, exposing the metal core. In Group 5, 10% of crowns underwent cohesive fracture, while 90% suffered adhesive fracture. In Group 6, 15% of crowns underwent cohesive fracture, whereas 85% were adhesive fractures.  Table 1 shows the fracture types obtained in each group and their relation to the core material and porcelain veneer type.

**Table 1:**
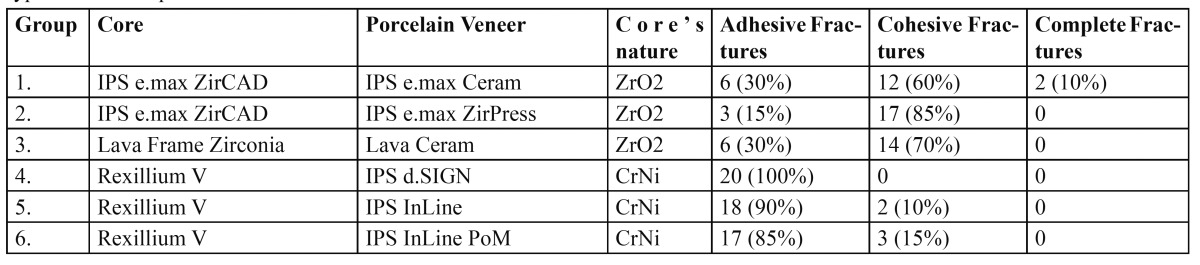
Description of the materials used in the six study groups and the type of fractures produced.

The zirconia-based porcelain veneer restorations (Groups 1, 2, and 3) showed a higher percentage of cohesive fractures (72%) in comparison to metal-ceramic crowns (8%) (Groups 4, 5 and 6); metal-ceramic restorations showed a higher percentage of adhesive fractures (92%) in comparison with zirconia-based porcelain veneer crowns (25%). Following Bonferroni bivariate analysis, statistically significant differences were found between groups, the incidence of cohesive fracture being greater for zirconia-based porcelain veneer crowns, while metal-ceramic restorations showed a higher incidence of adhesive fracture ( Table 2).

**Table 2:**
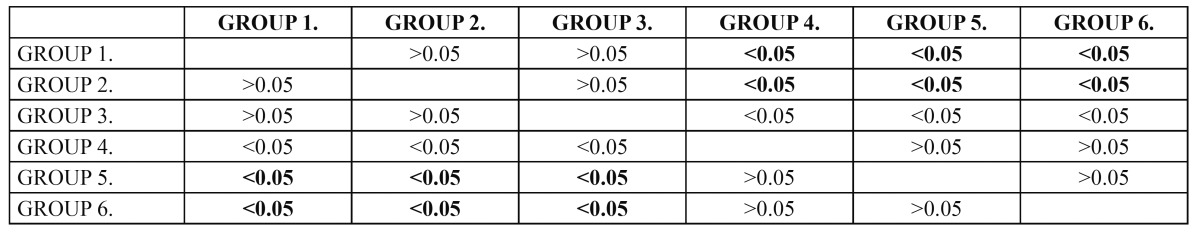
Bonferroni bivariate analysis.

**Figure 2:**
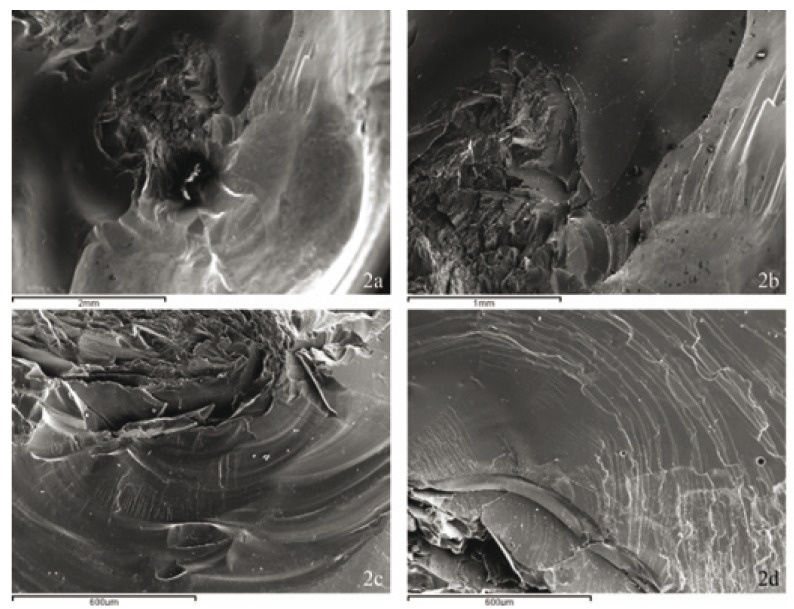
A.- Peripheral or radial surface fracture propagation adjacent to the area of contact with the antagonist (Specimen number 18, Group 1 SEM SE x25. B.- Same Specimen SEM SE x50. C.-The same fracture propagation pattern is seen in Specimen 8, Group 2, SEM SE x100. D.-The same fracture propagation pattern in Specimen 6, Group 2, SEM SE x100.

Surface fracture patterns for all the crowns analyzed at the point of occlusion with the antagonist were radial or peripheral, in other words, the deformation of the veneer material was produced in the occlusal zone, with the fracture projected from the central point of load application towards the periphery where porcelain delamination occurred (Fig. 2).

**Figure 3:**
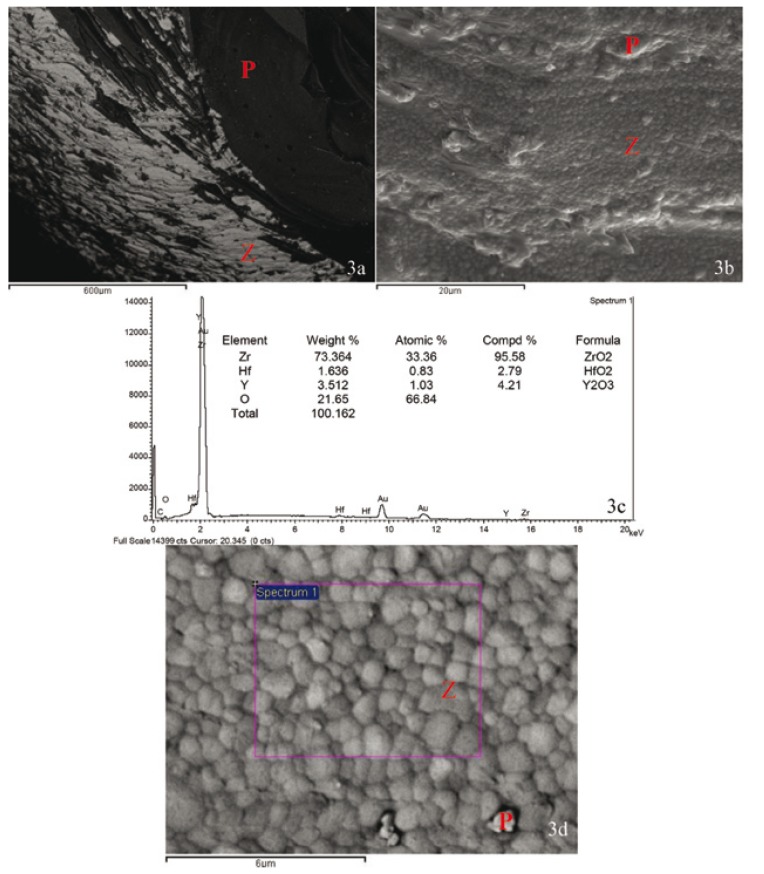
A.-Specimen 1, Group III 100x BSE. B.-Same Specimen x2500 SE. C.-Composition analysis from Spectrum 1 x10000 BSE. D.-Same Specimen x10000 BSE. P = Porcelain Veneer. Z = Zirconia

Composition analysis of selected specimens by SEM helped make a clear distinction between cohesive fractures and adhesive fractures (those that exposed the internal core) (Fig. 3).

## Discussion

*In vitro* tests aim to simulate clinical behavior and so aim to create an in vitro study model that recreates clinical conditions as precisely as possible. There are many characteristics of clinical situations that need consideration but are difficult to reproduce under laboratory conditions. Laboratory studies, particularly those involving static loading, provide limited predictions of the behavior that restorations might be expected to show when subjected to real clinical situations. Nevertheless, they can offer reliable results when specific factors are to be observed and compared in a controlled environment.

To date, little *in vitro* research has been carried out into the strength of porcelain veneers on zirconia-based ceramic restorations. Moreover, there are far fewer references to zirconia core restorations than to studies of the mechanical resistance of metal-ceramic crowns.

Numerous authors have performed static load tests to study the strength of ceramic materials in full coverage dental restorations (12-15) and compressive testing would appear to be an adequate method for evaluating resistance to fracture of crowns or fixed partial dentures (16). In the present study, the crosshead speed (0.5 mm/min) and static compressive load (5 KN) were established in light of a literature review dealing with these variables (12,17,18).

The full coverage restorations analyzed were fabricated on the basis of a master model in the shape of a maxillary first molar, as proposed by Agustín et al. (12).

From the present in vitro study, it can be confirmed that porcelain veneers with the same characteristics behave differently in response to static loading depending on the type of supporting core. For zirconia-based restorations, fractures occurred more frequently in the interior of the porcelain veneer. These results can be extrapolated to clinical practice given that, according to the literature, zirconia restorations fracture with greater frequency than metal-ceramic ones, with the main failure type being cohesive (chipping) (19). Stereomicroscope examination showed that 72% of fractures of porcelain veneers on zirconia restorations were cohesive (chipping).

Fischer et al. (19) studied zirconia-porcelain interfaces on zirconia crowns subjected to static loading to the point of fracture of the surface porcelain, carrying out SEM analysis of elemental composition and distribution. To date, no scientific evidence for a chemical bond between zirconia and the veneering ceramic has been found. The two materials appear to bond as a result of mechanical interlocking and through the formation of compressive strength resulting from thermal contraction during cooling following sinterization (20). In this way, Fischer et al. found that for zirconia restorations, the most frequent type of fracture occurred within the porcelain veneer rather than at the porcelain-zirconia core interface (19).

An important factor in full coverage restorations with zirconia cores is the morphology/design of its internal cap. Numerous authors insist on the importance of a design that follows an anatomical pattern. This will guarantee uniform thickness of the veneer porcelain over the entire crown and will avoid inadequate support of the porcelain resulting from incorrect cap design and reduce the likelihood of porcelain delamination (14,19,21,22). The morphology design used for each crown, regardless of whether the core is of metal or zirconia, was proposed by Molin et al. (13), who reported that an anatomical design of the internal cap can reinforce the porcelain veneer’s clinical resistance to fracture. A later *in vitro* study carried out by Kokubo et al. confirmed that the anatomical core model achieved higher levels of resistance to porcelain veneer fracture (14).

Despite having followed the indications put forward in these earlier studies, the percentages of cohesive fractures obtained with crowns with zirconia cores are high compared to metal-ceramic crowns. It may be that other laboratory procedures used during the zirconia processing might have a relevant influence on porcelain veneer behavior.

It is known that residual tensions of thermal origin are produced following the processing of zirconia-based porcelain veneer restorations. Nevertheless, to date there are no clear directives for the correct laboratory handling of the material, although Tan et al. (23) have shown that slow cooling and slow heating regimes used when firing porcelain to zirconia increase its fracture resistance. This laboratory procedure needs further investigation as it might help equalize the cohesive fracture resistance of zirconia crowns with that of metal-ceramic crowns.

The results of this preliminary study must be interpreted with caution since it is difficult to extrapolate in vivo clinical results from the static loading test alone. Further fatigue fracture testing in a wet environment and other clinical research is needed in order to confirm the results of the study.

## Conclusions

Within the limits of the present study, it was observed that:

1. Porcelain veneers show significantly different behavior in relation to the restoration core material. There is a higher incidence of cohesive fractures among zirconia-based porcelain veneer restorations. There is a higher incidence of adhesive fracture among metal-ceramic crowns

2. The fracture propagation surface pattern is of radial or peripheral type adjacent to the point of contact with the antagonist tooth, at the point of load application.
